# Blockchain-Based Context-Aware Authorization Management as a Service in IoT

**DOI:** 10.3390/s21227656

**Published:** 2021-11-18

**Authors:** Tidiane Sylla, Leo Mendiboure, Mohamed Aymen Chalouf, Francine Krief

**Affiliations:** 1 Laboratoire Bordelais de Recherche en Informatique, Bordeaux INP, CNRS, Université de Bordeaux, UMR 5800, 33400 Talence, France; francine.krief@bordeaux-inp.fr,; 2 Institute of Applied Sciences, University of Sciences, Techniques and Technologies of Bamako, Bamako, Mali; 3 COSYS-ERENA Lab, Université Gustave Eiffel, 33067 Pessac, France; leo.mendiboure@univ-eiffel.fr; 4 IRISA Lab, CNRS, Université de Rennes 1, UMR 6074, 22300 Lannion, France; mohamed-aymen.chalouf@irisa.fr

**Keywords:** Internet of Things, context-aware security, authentication, access control, ACE-OAuth, blockchain, smart contracts

## Abstract

Internet of Things (IoT) applications bring evolved and intelligent services that can help improve users’ daily lives. These applications include home automation, health care, and smart agriculture. However, IoT development and adoption face various security and privacy challenges that need to be overcome. As a promising security paradigm, context-aware security enables one to enforce security and privacy mechanisms adaptively. Moreover, with the advancements in edge computing, context-aware security services can dynamically be placed close to a user’s location and enable the support of low latency communication and mobility. Therefore, the design of an adaptive and decentralized access control mechanism becomes a necessity. In this paper, we propose a decentralized context-aware authorization management as a service based on the blockchain. The proposed architecture extends the Authentication and Authorization for Constrained Environments (ACE) framework with blockchain technology and context-awareness capabilities. Instead of a classic Open Authorization 2.0 (OAuth) access token, it uses a new contextual access token. The evaluation results show our proposition’s effectiveness and advantages in terms of usability, security, low latency, and energy consumption.

## 1. Introduction

The emergence of the Internet of Things (IoT) has enabled the development of new ubiquitous computing services involving users’ data and devices. However, the solutions proposed in this ecosystem raise security and privacy risks for users. Recently, the definition of solutions implementing user’s context-aware security and privacy mechanisms have been proposed [1,2]. These solutions use relevant context information to decide the implementation of security and privacy mechanisms tailored to the user’s current situation [3].

The security of IoT systems essentially concerns communications, data storage, and data access [4]. In this work, we are interested in the security of access to data stored in IoT applications and services. Authorization management mechanisms provide access control to protected resources. Although authorization management in IoT has been the subject of several works and standards, few of these works consider context-awareness in the implementation of this mechanism [1,5,6,7], and the decentralization of the authorization server [8,9].

The Internet Engineering Task Force (IETF) proposed a generic framework for the authentication and access control for the IoT, called Authentication and Authorization for Constrained Environments (ACE) using the OAuth 2.0 framework [5]. ACE-OAuth is based on Open Authorization (OAuth) 2.0 [10] and aims at allowing a third party to get access to the protected resources of a device called a resource server through Constrained Application Protocol (CoAP) [11]. In fact, the third party, called a client, must have an authorization token previously issued by the authorization server. The ACE-OAuth framework supports multi-dimensional IoT environments by preventing permission conflicts. For instance, the owner of a smart home and their partner can have concurrent access to the smart lock.

**Figure 1:**
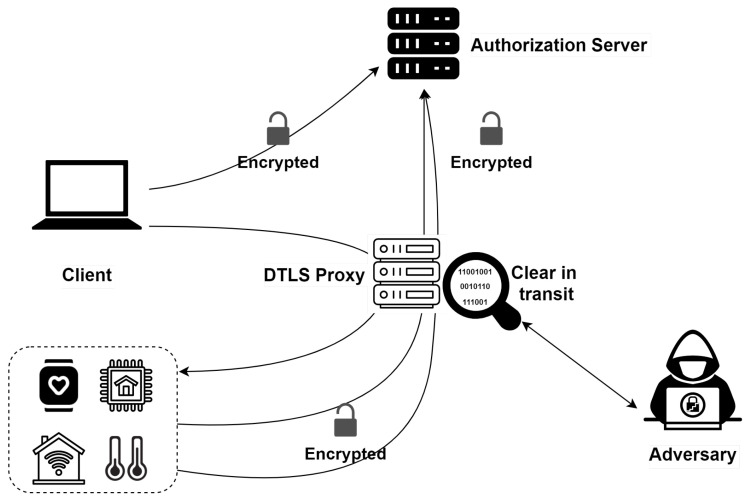
ACE Framework with DTLS security profile.

The client can use its authorization token to request access to the resource server’s protected resources using, for example, Datagram Transport Layer Security (DTLS) or Object Security for Constrained RESTful Environments (OSCORE) as secure communication channels. DTLS (Tschofenig and Fossati, 2016) and OSCORE (Selander et al., 2019b) are recommended security mechanisms in the ACE framework. They allow constrained devices to authenticate each other and perform secure communications. DTLS can be used to secure CoAP communications, whose exchanges are based on the User Datagram Protocol (UDP). The OSCORE protocol has been proposed to secure end-to-end CoAP exchanges that pass through intermediate proxies. Indeed, the information is not secured by DTLS during transit through intermediate proxies. A malicious proxy can modify the data in transit [12]. Figure 1 depicts the implementation of ACE with a malicious proxy.

However, ACE presents several limitations for the security of an IoT application. On the one hand, these applications are characterized by their dynamic environment. This dynamic characteristic makes it necessary to adapt authorizations dynamically in response to changing risks (caused, for example, by changes in the user’s context). For example, a doctor may have contextual permissions, allowing him to consult a patient’s health data from the hospital. However, he may not have permission to access the same data outside the hospital (e.g., from his home).

Another example is to give a patient the ability to assign exceptional and contextual access rights to his smart blood glucose meter data to a nurse (e.g., in the case of a critical situation at the hospital). In contrast, the nurse has limited access rights to the blood glucose meter data under normal conditions (a normal hospital situation). On the other hand, ACE security also depends on communications security (DTLS or OSCORE) and the authorization server. The authorization server centralizing all operations can represent a single point of failure because it can fail or be compromised.

In this paper, we propose a context-aware authorization management system based on the ACE framework. Indeed, this system will extend the ACE framework with context-awareness capabilities. This system can integrate with any context-aware security service for IoT (e.g., de Matos et al. [1], and Sylla et al. [2]). In ACE, clients must present a static access token containing permissions to the resource server. For supporting dynamic and contextual token-based permissions, we propose to condition access token generation on the validity of the user’s context. To mitigate the security risks of token transport in ACE, we propose using a new secure contextual authorization token.

Furthermore, as previously mentioned, a central authorization server can represent a single point of failure and can be compromised. Thus, to overcome this problem and strengthen the security of the proposed context-aware authorization management, we define a new blockchain-enabled decentralized context-aware authorization system based on the ACE framework. Indeed, the blockchain, whose trust and security are no longer to be proven, will allow the user to manage authorizations flexibly, dynamically, and securely without relying on a trusted third party [13]. Thanks to the proposed system, the user will be able to dynamically define the contextual access rights of each IoT application in a smart contract [14]. The smart contract will then generate the contextual access tokens upon request from the authorized clients.

Furthermore, our proposition implements the as-a-service approach. Thus it can be integrated into new network architectures (e.g., edge computing architecture). Our system allows the deployment of the context-aware security service and the blockchain in an edge computing infrastructure. This deployment has several advantages. First, the service can be deployed at any point in the network and thus always be placed close to the user. This enables real-time communications and reliable and efficient support of user’s mobility. In this paper, we suppose that user mobility support means the ability to execute and maintain services on edge computing nodes close to the user. Second, the proposed architecture is dynamic, flexible, and scalable.

The major contributions can be summarized as follows:

- A new blockchain-based decentralized context-aware authorization management system based on an extension of the ACE framework;
- A new secure contextual access token;
- Enhanced security and trust in the context-aware authorization through smart contract integration. The smart contract integration enables the user to be the central actor of the authorization management;
- Integration as a service within any context-aware security service in the IoT to enable dynamic and flexible authorization management. The as a service integration enables the proposed system to manage any other context-aware security service with high flexibility. Indeed, it is based on the as a service design approach;
- Deployment in new network architectures, including edge computing infrastructures to better support user mobility, reduce latency and enable dynamic, always-on security for IoT users. It can be location-aware and follow user mobility.

The rest of this paper is organized as follows. Section 2 compares the main works dealing with authorization management in IoT. Section 3 introduces the background of the main elements discussed in this paper. Section 4 presents the architecture of the proposed system, how it works, and its main advantages. Section 5 analyses and discusses the evaluation results and the security of this architecture. Finally, Section 6 concludes the paper and presents future work.

## 2. Related Work

Authorization management in an IoT environment has been the subject of several works. In this section, we analyze these works and identify their limitations.

An IoT environment is dynamic, characterized by frequent changes in the user’s global context. Therefore, Ramos et al. [15] proposed a context-aware security architecture for the IoT. This architecture includes a context-aware authorization management module. The proposed module is responsible for managing access control and capability-based authorization token management (issuance, revocation, etc.). In the proposed architecture, authorization management is centralized at the context-awareness management system. However, the proposed authorization tokens are not secure. These tokens are not encrypted nor signed when generated. Thus, an adversary can intercept and use a token issued to a legitimate client.

Sciancalepore et al. [6] proposed an adaptation of the OAuth 2.0 standard [10] for managing permissions and controlling access to resources in an IoT network composed of constrained devices. The resources of the devices in the considered network are cached at the gateway level. The gateway acts as a resource server and thus checks the access authorizations of client applications. The authorization tokens are issued by the authorization server after authentication of the client applications. However, the proposed system is not context-aware. In an IoT environment, the user context changes frequently, security risks evolve and authorizations adapt accordingly.

Claeys et al. [16] proposed an authentication and access control system based on the generic frameworks of ACE and OAuth1.0a. This system aims to manage and transport authorization tokens for constrained devices in an insecure network. The authorization tokens implemented are Proof of Possession tokens secured by COSE (CBOR (Concise Binary Object Representation) Object Signing and Encryption). As in [6], the tokens are secure, but they are not context-aware.

The work of [1,6,15,16] has focused on implementing centralized authorization management. This authorization management then has several limitations. The authorization server can be a bottleneck. It is also possible for an illegitimate authorization server to impersonate the legitimate server. Besides, centralized authorization management in IoT applications poses the problem of user trust in a third party. The authors of [17] proposed a decentralized and secure end-to-end authorization management system based on Object Security Architecture (OSCAR) [18] and ACE, called IoTChain. The Blockchain replaces the authorization server of ACE. The OSCAR architecture, an enhancement of DTLS, is used for key management and secure authorization tokens and data exchange. However, similar to [6,16] this system is not context-aware.

Blockchain is a disruptive technology that enables realizing reliable transactions built on a decentralized and implicit trustworthy system. Thanks to smart contracts, users can edit and automate the rules of transactions. Recently, many researchers have focused on how blockchain technology can improve IoT security. In this context, Xu et al. [19] proposed a blockchain-based access control scheme for solving a single point of failure and bottleneck problems of central authorization servers in large-scale IoT systems. The proposed solution is scalable and supports heterogeneous devices. However, it is not adapted to daily users’ IoT environments, for example, smart homes or e-health systems.

In the same context, Al Breiki et al. [20] addressed the data access control problem in IoT. To this end, they proposed a decentralized IoT data access control scheme based on blockchain technology. This proposed scheme presents the advantage of supporting several data storage services. Nevertheless, the authors have not addressed the access rights management entities (administrators and end-users): grant, revocation, and suspension.

Zhang et al. [21] proposed a blockchain-based distributed and trusted access control system. This system includes multiple access control smart contracts, a judge smart contract, and a registration smart contract. This system can statically or dynamically validate accesses and can penalize nodes responsible for bad behavior. However, this system is not context-aware. Indeed, the access validation does not take into account the context of the users. Furthermore, the proposed solution is not flexible enough, does not perform well enough (latency), and does not support user mobility. Furthermore, the solutions proposed in these works ([19,20,21]) are not context-aware, i.e., they could not dynamically manage access control according to users’ situations (see Section 3.1).

Arfaoui et al. [8] proposed a Context-Aware Attribute-Based Access Control (CAABAC) approach. The proposed solution considers context information as well as user attributes in access control. It also integrates Ciphertext-Policy Attribute-Based Encryption to ensure data confidentiality. This solution has several advantages. It uses context to adapt access control to the resources of constrained devices dynamically. It solves the problem of the trusted third party of CP-ABE (Ciphertext-Policy Attribute-Based Encryption). Finally, it enables the protection of the user’s data. However, this solution is not decentralized and relies on two systems (the Key Generation Center (KGC) and the Attribute Authority (AA)), which can be complex to manage for a lambda user. Besides, similar to [21], user mobility is not supported, and scalability is not guaranteed. Indeed, the scalability of the solution has not been evaluated.

Mehta et al. [22] addressed the single point of failure problem of central authorization management in context-aware IoT applications. They proposed a decentralized context-aware access control model. The proposed access control model leverages context-awareness to provide access to IoT applications only based on the proper context in a decentralized manner. Nevertheless, the proposed system is vulnerable to several attacks targeting decentralized systems, for example, the Sybil attack.

Song et al. [23] tackled the centralization of context-aware access control in cross-domain and distributed pervasive computing. The authors proposed a context-aware cross-domain access control scheme for such an environment. The proposed scheme helps to solve context-aware authorization in a cross-domain IoT system that uses a peer-to-peer method to construct an access request decision tree. However, even though the scheme uses cooperative access control decisions among resources servers, it relies on a central authorization server. As denoted by Mehta et al. [22], a central authorization server can be a single point of failure.

However, as seen in Section 1 and mentioned in [2,3], context-aware security can be a solution for many IoT applications such as e-health. Moreover, authorization management and access control should consider user preferences, context, and context-related risks. Table 1 summarizes the comparison of the reviewed works dealing with decentralized and/or context-aware authorization management in an IoT environment. These works are hardly practicable within the context of IoT environments addressed in this paper, such as smart home and building and e-health systems. Indeed, the users are not a central actor of the authorization management in these schemes. The ACE framework proposed by IETF is a standard draft authentication and authorization scheme for IoT. Indeed, it enables both authentication and access control services for IoT applications and devices. To the best of our knowledge, the idea of dynamic and blockchain-based decentralized context-aware authorization management in IoT based on the ACE framework has not been proposed yet.

This work presents two main innovations. First, it enables the ACE framework to support the dynamic aspect of most IoT environments, considering the users’ situation in managing authorizations. Second, blockchain technology integration enables the users to directly manage authorizations using a decentralized approach without going through a trusted third-party authority. Thus, in this paper, our goal is to demonstrate the advantage of such an approach (security, context-awareness, dynamicity, and scalability) for authorization management in IoT applications. To this end, the proposed architecture is presented in Section 4. Since this architecture implements token security and uses blockchain technology to ensure scalability, the following section describes these two concepts.

Furthermore, the architecture is designed following the ‘as a service’ approach. The ‘as a service’ approach allows meeting the challenges of supporting numerous IoT applications and the necessary integration in new network architectures, especially Edge Computing infrastructures.

## 3. Background

In this section, we first review the ACE framework token security, then describe context-aware security and blockchain technology.

### 3.1. Context-Aware Security

A Context-aware IoT system uses relevant contextual information from the user’s devices (e.g., weather, ambient temperature, and light, proximity to other devices, geographic location, etc.) to perform automatic and dynamic adaptation actions [3]. Context-aware security, a sub-field of context-aware computing, is a security paradigm that addresses many security challenges in ubiquitous computing systems such as the IoT. For example, a major challenge among these security challenges is the automatic collection, processing, and exchange of user information by various applications. Reliable and secure context-awareness management is the cornerstone of a context-aware security system.

Context-awareness is the process that aggregates, evaluates, processes, and merges contextual information from multiple sources to determine the user’s context. This process is detailed in several work [3,24]. For example, in Sylla et al. [2], a context-aware security and privacy protection architecture including a knowledge plane has been proposed. This knowledge plane handles context determination and prediction using artificial intelligence techniques. It also has several benefits such as contextual risk assessment, users’ security, and privacy preferences management. For example, a context critical situation can be determined from information such as user location (hospital) and his vital signs, e.g., no motion and very low heart rate.

An IoT context-aware security system should be based on secure and trustworthy context-awareness management [3]. Indeed, falsified contextual information can be used to mislead the security level adaptation. Using secure and reliable context information collected from trustworthy context sources can help user’s trust in the context-aware security adaptation. For example, in Sylla et al. [4], we addressed these challenges by proposing SETUCOM (Secure and TrUstworthy COntext-awareness Management).

### 3.2. ACE Tokens Security

OAuth2.0 is a protocol that allows a user to grant a third-party web application permission to access its protected resources without necessarily revealing its credentials [10]. The authorizations are granted to third-party web applications, referred to as clients, through tokens. Access tokens in OAuth 2.0 are exchanged in the JSON Web Token (JWT) format, enabling specific information for authorization requests (client identity, authorized accesses, etc.). The JSON Object Signing and Encryption (JOSE) format has been proposed for securing tokens. This standard includes several specifications for encryption, signing, and integrity of JWT tokens [25,26,27,28]. However, since OAuth 2.0 is not suitable for the IoT constrained devices (CPU, memory and energy), the ACE framework was proposed.

To address the requirements of constrained devices, including IoT devices in ACE, the Concise Binary Object Representation (CBOR) format has been proposed [29]. This format’s goal is to facilitate the definition and the transmission of small messages for IoT applications. Secure tokens generated according to the ACE framework are thus represented in the CBOR Web Token (CWT) format [30]. The COSE standard was defined to secure CWT tokens similarly to JOSE for JWT tokens. The main difference between COSE and JOSE is the concise and compact CBOR format for COSE, while JOSE uses the JWT format. Thus, in our system, we propose to use COSE as it is secured and suitable for IoT applications. The implementation of COSE is detailed in Section 4.5.1.

**Figure 2:**
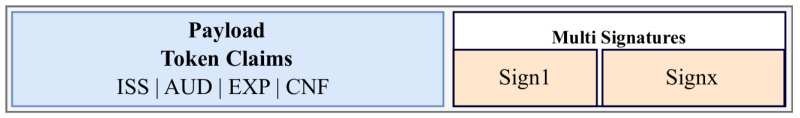
A COSE Proof-of-Possession token format.

Proof of Possession (PoP) is an access token extension designed to enable secure association of tokens and requests allowed by those tokens [31]. A cryptographic key is associated with the token and allows the client to demonstrate possession of the associated secret when accessing a resource. The receiving resource server verifies that the key used by the client is the one associated with the token. The key associated with the token can be a symmetric or asymmetric cryptographic key. The difference between these two approaches consists in the choice of the cryptographic algorithm used for key generation [5]. The symmetric PoP key can be randomly generated by the client and be included in the token. This key can be used later for introspection. For the asymmetric PoP key, the client generates a key pair and sends the public key to the authorization server. The Figure 2 illustrates a PoP token format. The payload consists of four fields. ISS (issuer) identifies the issuer of the token. AUD (audience) identifies the recipients of the token. Thus, each client must be identified to the AUD to process the token. EXP (expiration time) represents the validity period of the token. CNF (confirmation), a JSON object identifying the PoP key is used to establish PoP [32].

### 3.3. Blockchain

**Figure 3:**
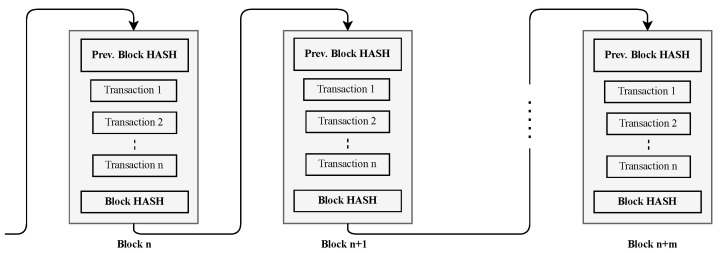
A blockchain diagram.

Blockchain is a distributed, replicated, and permanent ledger in which records are time-stamped and signed blocks [14,33]. A block is identified by a hash resulting from a cryptographic hash process. The hash of each block points to the hash of the previous block, and so on. This referencing of blocks hashes forms a chain of blocks, resulting in the name blockchain. Figure 3 shows a blockchain diagram.

The Merkle Tree is a data structure containing the hash of all transactions in a block. The Merkle Root of a block is created from the fingerprint of each transaction pair. A single bit in a leaf of the Merkle tree cannot be changed without altering the Merkle Root. Thus, the block is unalterable. Each member of the network, called a node, has a complete copy of the chain. Each time a node makes a transaction, a miner adds the transaction to a block and then validates that transaction. A new block is only added to the chain if certified by a distributed validation algorithm called consensus. The well-known consensus algorithms are Proof of Work (PoW), Proof of Stake (PoS), and Practical Byzantine Fault Tolerance (PBFT).

The security of exchanges in a blockchain network is ensured with the use of asymmetric cryptography. Each user interacts with the blockchain through a public/private key pair. The public key is used to identify a user, while the private key is used to sign transactions. Thus, a blockchain network implements common security functions (authentication, confidentiality, integrity, and non-repudiation) through asymmetric cryptography. It can be noted that transactions are stored in plaintext in the blockchain ledger. Therefore any user can read and verify all transactions made over time (transparency) even if pseudonyms are used to protect users’ privacy. The blockchain decentralized architecture (peer-to-peer network) is based on reliable consensus algorithms that guarantee a high replication level of the data (scalability). Thus, blockchain represents an alternative security solution (transparent, scalable) that works without a central control authority.

Another element characterizing the blockchain is the smart contract. The smart contract is a computer program stored in the blockchain enabling code execution on demand and under certain conditions (similar to written contractual clauses) [13]. For example, the condition for executing a function in a smart contract may be the context, e.g., “the user is in the hospital”. It automates transparent exchanges at a low cost for the stakeholders without the intervention of a trusted third party. For this purpose, a smart contract provides several functions, also called Application Binary Interface (ABI), to interact with it.

## 4. System Architecture

**Figure 4:**
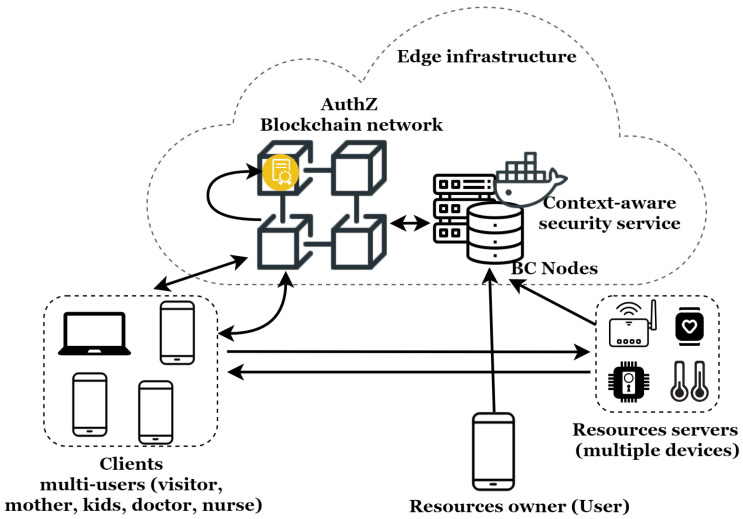
Blockchain-enabled decentralized context-aware authorization management architecture in the IoT.

This section introduces the proposed system, details its operation, and presents its main benefits. Figure 4 illustrates the architecture of this system as well as the exchange flow for secure IoT resource access for users.

We propose a novel blockchain-enabled decentralized context-aware access control system based on contextual access tokens. The architecture is composed of the ACE Framework’s elements, a decentralized authorization blockchain network, and a context-aware security service for the IoT [2]. This dynamic and flexible context-aware security service, designed following the ’as a service’ approach, can be dynamically placed at any point of an edge infrastructure. The integration as an edge service enables very low latency communication (necessary for real-time services) and better support for user mobility.

According to ACE terminology, the elements of our architecture are:

– **Resources’ owner:** Legal owner of the resources protected by the resource servers, such as the IoT application’s user (e-health, connected home, etc.);
– **Resource’s server:** Stores and manages the protected resources. In our context, these are composed of IoT devices, such as temperature sensors, glucose meters, smartwatches, locks, etc;
– **Client:** Party that needs to access the resources protected by the resource server. This is usually an application in which there is a user behind. For instance, in multi-user environments, a client can be the user’s wife, kids, and or temporary hosted person. It can also be the doctor, nurse or electricity technician;
– **Context-aware security service:** Receives context information and determines the user’s context. It is responsible for the dynamic adaptation of the security mechanisms according to the user’s context. Indeed, this is done by dynamically deploying security and privacy mechanisms according to the user’s situations;
– **Authorization blockchain network:** Depending on the resource owner’s decisions, it generates the contextual authorization tokens used to grant authorizations to clients. Valid contextual access tokens enables clients to access protected resources;
– **Blockchain node:** the authorization blockchain network member.

We assume that constrained devices (e.g., Raspberry Pi Zero w and Raspberry Pi 3B) can perform lightweight asymmetric cryptography operations, have root certificates, and can contact a certification authority. Constrained devices that are not able to do that must use a proxy that could perform these operations. Therefore, the concerned devices (resource servers) will sign and verify the identities linked to the signatures.

### 4.1. Threat Model

A threat model provides a way to model the threats and adversaries to which a system may be exposed. Analyzing the various threats and adversaries is a critical step in designing a security and privacy system. The proposed system is exposed to several threats:

– **Contextual access token integrity**: Contextual access tokens must be protected from forgery (illegitimate creation) and unauthorized modification. Their validity contexts must also be protected against forgery and modification (see Section 5.3). A mechanism must be provided to detect stolen or illegitimate context access tokens;
– **Communications security**: The proposed access control mechanism is based on contextual access tokens. It is crucial to protect the contextual access token exchanges against the Man-in-the-middle attacks (e.g., eavesdropping, tampering). These attacks can lead to token forgery or modification;
– **Privilege escalation**: Privilege escalation occurs when a malicious user exploits a system vulnerability to access resources ordinarily unavailable to him. Privilege escalation attacks target many IoT access control systems. Privileges escalation can occur when an attacker takes possession of an authorized user’s devices. Therefore, the proposed system must enforce privilege escalation mitigation mechanisms;
– **Context-aware security service**: Generates and manages validity contexts according to the resource owner’s preferences (e.g., what a user can access and in which conditions he can access the door lock). It must be protected against attacks on its availability. Validity contexts must remain protected if the context-aware security service is compromised;
– **Resources servers**: They are constrained and subject to several physical and network attacks. They must be secured to protect context access tokens during processing and storage;
– **Resources owners**: They may or may not be security-aware users. They must be aware of the importance of security in their interactions with the context-aware security service. His interactions with this service must be performed on secured communication channel. It must also be protected against identity theft. Indeed, this consists in protecting the user’s private keys;
– **Clients**: they are heterogeneous (laptop, smartphone, tablet, etc.) and store contextual access tokens. They must use secure communications. The contextual access tokens created must be protected against theft and clients prevented from forging valid tokens.

### 4.2. Main Advantages of the New System

Blockchain offers several advantages in terms of security, trust, and performance (decentralized architecture) [34]. Edge computing deployment of a context-aware security service also provides several benefits and opportunities for context-aware security and privacy in IoT. In this sense, it provides better user mobility management, and guarantees better performance (latency and throughput), flexibility, and scalability [2]. Therefore, integrating blockchain and context-aware security service is an attractive solution for decentralized context-aware authorization management in IoT. Indeed, the blockchain network and the user’s context-aware security service could be hosted in the edge infrastructure. This eliminates latency issues due to communications between that service and the node, the entry point to the blockchain network. Furthermore, our proposal, based on ACE OAuth2 framework (ACE-OAuth), manages as well multi-users and multi-devices IoT environments [5]. Indeed, it avoids wrong permissions enforcement and conflicts (see Section 4.4). The smart contracts enable concurrent access to a resource, for instance, a contextual access token.

### 4.3. Authorization Flow

**Figure 5:**
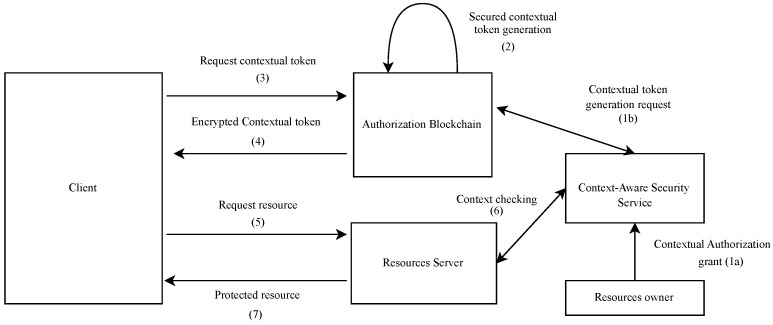
Proposed framework detailed flow.

Figure 5 illustrates the different flows of the proposed architecture. In the following, we assume that the user is previously registered with the Authorization Blockchain. The user’s devices are also pre-registered with the context-aware security service. We have done similar work in [4] regarding the secure management of the user’s devices with a context-aware security service. These registration steps are not discussed in this paper. The proposed authorization flow starts with the user preferences specification (step 1a). This is performed via the context-aware security service. This enables the user to specify at any time which applications can obtain contextual access authorization, which resources they can access, and under which conditions they can obtain these authorizations. The user’s context-aware security service contacts the smart contract with the data needed to generate the contextual access tokens (step 1b). Only the user can order the generation of contextual access tokens.

The smart contract executes the “Generate contextual access token” function and dynamically generates the secure contextual access token (step 2). The “Generate contextual access token” function is similar to the token endpoint of the ACE framework’s authorization server. The proposed tokens have the format of the ACE framework’s authorization tokens and add support for context-awareness. We propose to bind, to a token, the identifier of its validity context. This allows the smart contract to check the validity of the token through the context-aware security service.

**Figure 6:**
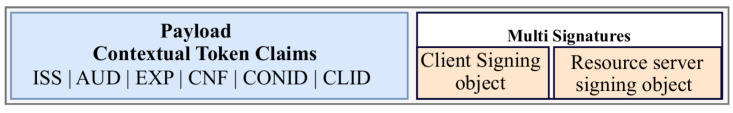
Proposed contextual access token, with *conid: hash of the context identifier, clid: hash of the client identifier*.

The token’s validity period is defined when the token is issued and depends on the use cases. In general, the period of validity of OAuth 2.0 access tokens may vary between 1 and 60 days [35]. Thus, each contextual access token includes the hash of its valid context identifier. In addition, similar to ACE, the proposed contextual access token also includes the hashes of the client’s and resource server’s public keys and two digital signatures generated by the smart contract intended for the client and the resource server (here, the IoT device). This protects the contextual access token from modification and enables the receiving entities (client and resource server) to ensure that the contextual access token was generated in the blockchain. Figure 6 represents the extension of the PoP token into the proposed contextual access token.

The proposed token provides perfect forward secrecy. The perfect forward secrecy allows the encrypted data to remain confidential if a previous key used to encrypt the data is revealed. Indeed, we use the Ephemeral Diffie-Hellman Over COSE (EDHOC) for token payload security. The security of the contextual access token is discussed in Section 4.5.1. In addition to the “Generate contextual access token” function, the smart contract functions are listed in Table 2.

The client can only call the “Request contextual access token” function. The other functions are reserved for the user’s context-aware security service.

A client aiming to access protected resources must contact the user’s smart contract (step 3). It calls the “Request a contextual access token” function of the smart contract. The smart contract signs the token and delivers it to the client (step 4). When the client receives the token, it verifies the signature of the smart contract before decrypting it. The client presents this contextual access token to the resource server (step 5). The communication between the client and the resource server security and the token security are discussed in Section 4.5.2. There are two possible cases: the device can communicate directly with the client (case 1) or the resource server is highly constrained and must go through a proxy to communicate with the client (case 2).

The second case will not be considered in this paper. When the device can communicate directly with the client (first case), the resource server must authenticate the client and validate the token in two steps. The resource server must authenticate the client before any operation. First, it verifies the signatures of the smart contract and the client. Then it checks the token’s lifetime. If the token is still valid, it checks the proof of possession (PoP) of the token by the client. When the PoP is established, the resource server uses the token’s context identifier to verify the match between the user’s context and the context of the access token. This verification is done with the context-aware security service (step 6). This context check is performed to dynamically allow/deny access depending on the user’s context. For the nurse case, (“critical situation in the hospital”, i.e., the user is located at the hospital, and the monitoring of his vital signs shows a very degraded situation), if the context check has given a valid answer, then the patient’s (user’s) glucometer gives access to the client application (the nurse’s application). If the token context is valid, the client can access the protected resources (step 7).

### 4.4. Multi-Users and Multi-Devices Environments

IoT environments are often multi-dimensional. They can include multiple users and multiple devices. For instance, there can be two parents, kids, and temporal visitors in a household in a smart home environment. In e-health applications, there can be several doctors and nurses, hospitals, and insurance companies. Therefore, an IoT authorization management framework design must take into consideration this multi- dimension. Designing an authorization framework for such an environment is a challenging task [36].

Figure 4 illustrates the system within a multi-dimension IoT environment. The proposed system in this paper is designed based on the ACE-OAuth standard. Utilizing multiple contextual access tokens for the same client (user) and resources server (device) can lead to permission conflicts. To avoid these conflicts, the resources server stores only one PoP key per token [5]. Every additional token linked to the same PoP key will update the existing token with the corresponding permissions.

More specifically, our system clears conflict scenarios by using priority-based access policies. To that end, the user defines access priorities when setting authorizations. When two users try to access a device simultaneously, the user with higher priority will be authorized, and the other one, with lower priority, will be blocked. It is possible for two users with equal priorities try to simultaneously access a device at the same time. In this case, our system uses mutex semaphore technique to clear conflict [37]. The conflict is avoided by performing the mutual exclusion of the users while accessing to device. Indeed, when a first user accesses the device, the second will be excluded. For example, when two parents try to access the thermostat setting simultaneously, the first parent will be authorized, and the second will be blocked.. Thus, our system manages well the multi-user environments.

Smart contracts can manage multiple access to the same resource. Nevertheless, in our proposition, every client can access only the token generated for him. This restriction is made possible by the inherent properties of smart contracts [38].

The access privileges delegation of our system is managed through the proposed secure contextual access token. Each secure contextual access token is generated on the owner (administrator) demands and only for one user. The contextual access token contains the scope that delegates a set of privileges to a specific user. The context (e.g., situation: location, time, and conditions) defines the validity of this scope. Furthermore, when user’s privileges are not good, the administrator can easily revoke the underprivilege token and generate a well-suited privilege one. The process is the same for an over-privileged user.

**Figure 7:**
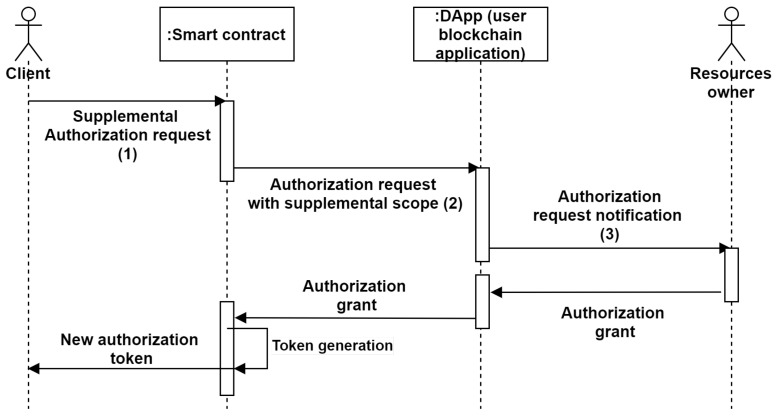
Supplemental authorization request flow.

Furthermore, when a user wants more privileges, our system allows this user to make the supplementary privileges delegation request. The supplemental privileges request is managed by calling the ‘Upgrade a contextual access token’ of the smart contract. Indeed, the administrator (resources owner) operates only for request validation or not. Thanks to our system based on ACE-OAuth, the owner’s credentials are not revealed during the process. Thus, the proposed system usability is enhanced. The supplemental authorization request in our system process is illustrated in Figure 7.

### 4.5. Secure Contextual Access Tokens and Secure Token Exchange

This section introduces contextual access tokens’ security (generation, use and exchange between authorization server, clients and resource servers).

#### 4.5.1. Secure Contextual Access Token

The contextual access tokens generated by the smart contract are in JSON Web Token (JWT) format. When the client decrypts the token, it must put it in COSE format before presenting it to the device. This format change is due to the need to adapt the authorization token to the constrained device. During the contextual access token generation, the smart contract encrypts the JWT object using the recipient client’s public key and signs it. The signature is performed with the Elliptic Curve Digital Signature Algorithm (ECDSA) [39]. In addition, only a client authorized in advance by the smart contract can call the contextual access token request function. Upon receipt, the client verifies the signature of the smart contract and decrypts the token. When the client successfully decrypts the token, it encodes this last in COSE format, adds its signature, and transfers the COSE object containing the encrypted contextual access token to the resource server. To do this, it establishes secure communication with the resource server.

On receiving the token, the device checks the client’s signature and decrypts it if the signature is valid. The device then verifies the signature of the smart contract associated with the token and proceeds to verify the proof of possession (PoP) of the client’s token. If the smart contract signature is not valid or the PoP is not established, it rejects the token.

#### 4.5.2. Secure Communication

The communications between the resource owner and the context-aware security service and between the client and the blockchain will use Transport Layer Security (TLS) channels. This can be explained by the sufficient capabilities of the devices running the resource owner and client applications (e.g., smartphones).

The communication between the client and the resource server can cross a multitude of unsecured underlying networks. An interesting alternative to transport layer security is application layer security. Indeed, application data security is particularly well suited to the IoT, requiring few resources to implement. Thus, as expected in the ACE framework, we use Ephemeral Diffie-Hellman Over COSE (EDHOC) [40] for secure token exchanges between the client and the resource server. The interesting properties of EDHOC justify this for the security of COSE token exchanges. Indeed, EDHOC allows for a secure and mutually authenticated communication channel suitable for constrained devices [40].

### 4.6. Dynamic Revocation of Clients and Tokens

Another major advantage of our proposition is the dynamic revocation of authorization tokens or authorized entities by the user. Thus, our proposition implements the user-centric approach.

#### 4.6.1. Clients

Client revocation is dynamically performed according to the user’s preferences (e.g., temporary contextual access token) defined through the context-aware security service. When a user aims to revoke the permissions of a client, the context-aware security service calls the “Revoke Client” function of the smart contract. The user performs this process through an easy-to-use application. Subsequently, the client’s public key is added to the list of revoked permissions. The contextual access tokens generated for that client are invalidated and are no longer accepted by the resource server.

#### 4.6.2. Contextual Access Token

The resource owner may decide to revoke a valid contextual access token assigned to a client. This process is launched manually (i.e., on the user demands) and performed automatically by the context-aware security service following the smart contract. For example, when the user’s context changes (the patient is no longer in a critical situation if we use our example from the beginning of this paper, i.e., improvement of his vital signs), the exceptional contextual rights that were granted to the nurse must be revoked. To do so, the user sends (through the context-aware security service application) a token revocation request to the context-aware security service. The context-aware security service calls the smart contract function “Revoke contextual access token”, which will immediately invalidate the token. This function takes the ID of the token to be revoked as an argument. It checks the token’s existence in the database associated with the smart contract. It then changes the status of the token (revoked). Once this operation is completed, it will no longer be possible to use this token.

## 5. Performances Evaluation

In this section, we evaluate the performance of the proposed solution. First, we describe the experimental environment and the considered scenario. Then, we evaluate the feasibility of our approach. Next, we discuss the obtained results. Finally, we analyze the security of our system.

### 5.1. Experiments Environment Setup

The objective of this experiment, considering a realistic IoT architecture (IoT devices and edge nodes), is to demonstrate that our solution, which extends ACE, presents acceptable performances. Indeed, the two proposed improvements (underlying architecture and context-adaptation) could have a significant impact in terms of performance (communication/calculation overhead).

To prove the feasibility of the proposed solution, we considered the e-health scenario mentioned as an example earlier in this paper. The solution is also valid for many other IoT applications such as smart home/smart grid (access to an electric meter), smart fitness, etc. Therefore, this scenario can be seen as a generic scenario. The user has a smart glucose meter (resource server) that is deployed in our experiment on a Raspberry Pi Zero w with a Lite Raspberry PI operating system, to evaluate our proposal in a real-life IoT environment. The context-aware security service is hosted as a virtual machine (also a test blockchain node) on a machine with the Ubuntu 20.04 LTS operating system. This machine is equipped with a 2.7 GHz dual-core Intel Core i7-6820HQ processor and contains 16 GB of RAM.

For the proposed decentralized authorization server, we used two implementations of blockchain technology: Hyperledger Fabric (v1.4.10) [41] and Ethereum (Go Ethereum v1.10.1) [42]. These platforms were chosen for two main reasons. First, these platforms are frequently used in the literature for the evaluation of blockchain-based solutions. Additionally, they enable the implementation of smart contracts, a required element for our authorization system. During the experimentations, we considered permissioned networks (where the identity of the nodes is verified) composed of eight blockchain nodes involved in the data verification process.

Similarly, it should be noted that the smart contracts have been implemented with Hyperledger Fabric and Ethereum. Therefore, the differences that can be observed in Section 5.2 are only due to the performance of these two blockchain implementations. To compare the performance of these blockchain-based solutions, we also used a Python implementation (“HappyEmu”, 2018) of a locally deployed centralized ACE-OAuth 2 authorization server as a basis for comparison. The defined smart contracts and the scripts used in the implemented environment, for the evaluation of the results are available online [43].

### 5.2. Results

This section presents and discusses the results of the performed evaluations, i.e., communications security and decentralized authorization management in the blockchain.

#### 5.2.1. Communications Security

To demonstrate our solution’s feasibility and benefits in terms of security, execution time, and energy consumption, we compared it with two authorization management approaches based on CoAP without security and CoAP DTLS, respectively. To do so, we used the LibCoAP library [44] for IoT with a 2048-bit RSA (Rivest Shamir and Adleman) certificate. In the rest of this paper, we will refer to the use of CoAP without security as simple CoAP.

DTLS is widely used for implementing secure CoAP-based communications in the IoT. However, it requires a 341-byte overhead in the best case, i.e., with a prior distribution of cryptographic keys (pre-shared keys) [45]. In contrast, EDHOC requires a maximum overhead of 97 bytes with a pre-distribution of keys. Therefore, our EDHOC-based solution requires much less bandwidth than DTLS to perform secure communication (access to secure device resources).

**Figure 8:**
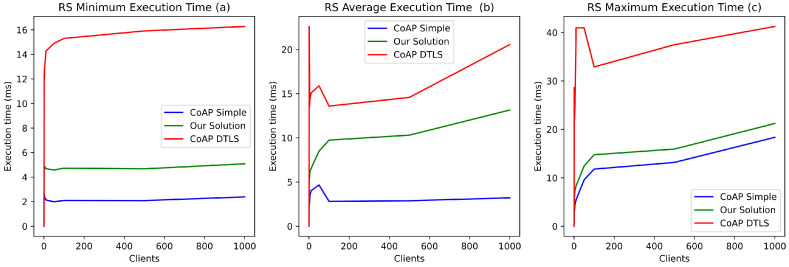
CoAP Server response execution time on the resource server.

Furthermore, real-time or near-real-time processing is required for e-health applications (e.g., for vital signs monitoring). Therefore, authentication and access control mechanisms should not exceed a few milliseconds. The establishment of a DTLS channel (DTLS Handshake) significantly impacts performances by greatly increasing the processing time of the resource server (medical device), whether in the best case (Figure 8a) or the worst case (14 milliseconds with 5 simultaneous requests, 30 milliseconds with 1000 simultaneous requests) (Figure 8c). In contrast, the use of EDHOC reduces this impact on resource server performance. It increases latency slightly by 5 milliseconds with the same number of concurrent requests (Figure 8b). Figure 8 illustrates the comparison of CoAP without security, CoAP DTLS, and our solution. The evaluations also show that our proposition better supports the simultaneous processing of a large number of client access requests to protected resources using contextual access tokens.

**Figure 9:**
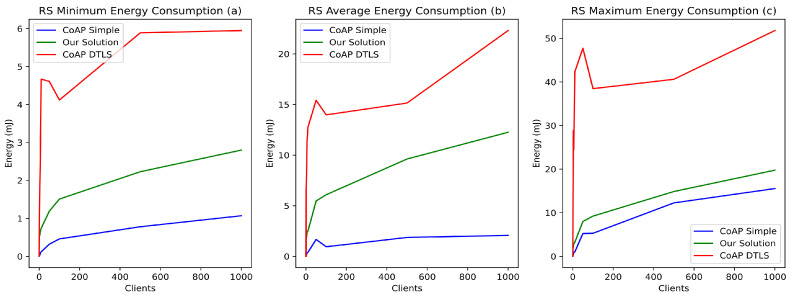
Impacts of simple CoAP, CoAP DTLS, and EDHOC on RS energy consumption.

In the considered application scenario, the vast majority of the devices have a low-capacity battery (e.g., AA or AAA 1.5-volt batteries). Therefore, energy consumption is an essential factor to consider when implementing suitable security mechanisms. Thus, we evaluated the energy impact of our proposition compared to CoAP and CoAP DTLS. Figure 9 illustrates the impact of these mechanisms on energy consumption. The results show that, on average, our solution increases energy consumption by 4 to 11 mJ compared to simple CoAP, while CoAP DTLS increases energy consumption by 15 to 25 mJ depending on the number of concurrent requests (Figure 9b). The maximum energy consumption induced by our solution is slightly higher than simple CoAP, by about 2 mJ. However, the impact of DTLS on energy consumption is much higher than our solution, by more than 20 mJ (Figure 9b). Therefore, our proposition has a low impact on the energy consumption of devices and is suitable for IoT, ensuring a very good balance between security and energy consumption of the nodes.

#### 5.2.2. Decentralized Authorization Management

We aimed to demonstrate the benefits of our solution for authorization in the ACE framework and, in particular, to evaluate the performance of the system architecture that we proposed (cf. Section 4). To do so, we compared the duration of contextual access tokens’ generation in a centralized (ACE-OAuth2 authorization server) and decentralized (Hyperledger Fabric, Ethereum) approach for a variable number of concurrent clients (200–1000). For the sake of this experiment, we utilized the following contexts: the night at home, the day at home, and the day at work between 8 a.m. and 4 p.m. at the hospital.

**Figure 10:**
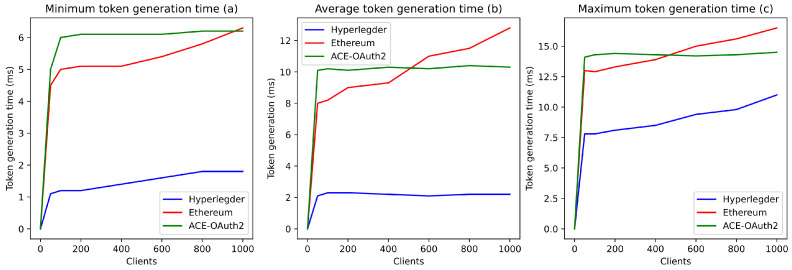
Token generation time by the blockchain and a Classic ACE-OAuth server.

Figure 10 shows that a centralized approach offers a significant level of scaling. Indeed, whatever the number of concurrent clients considered, the contextual access token generation times (average, minimum, maximum) remain constant (respectively about 10, 6, and 14 ms). This generation times is a clear advantage over an Ethereum-based decentralized authorization server, which performs better for a low number of clients (below 500) (Figure 10a.) but worse for a higher number of clients. Similarly, the centralized approach guarantees, compared to Ethereum, a lower maximum generation time (gain of more than 2 ms) (Figure 10c). Thus, the current implementation of the Ethereum blockchain demonstrates its limitations in this context. The consensus process (Proof of Work) and the query processing procedure (low parallelization) currently used by this technology lead to longer delays than the centralized approach for a high number of (simultaneous) clients.

However, we can also see in Figure 10 that the Hyperledger-based implementation guarantees better performance than the Ethereum-based one and the centralized solution. Indeed, as with the classical approach, the Hyperledger-based solution guarantees a constant average generation time, regardless of the number of clients (Figure 10b). Moreover, the average generation time is about 4 to 5 times lower than the centralized solution. In addition, the minimum and maximum generation times offered by this approach are much lower than those allowed by the classical approach (about 4 ms lower on average for a number of clients between 200 and 1000). Therefore, this Hyperledger implementation seems to demonstrate the benefit of a decentralized authorization server for the ACE framework.

Moreover, with our solution, authorization servers could be deployed at the edge of the network. As demonstrated by the authors of [46], with an edge deployment, latency could be reduced by several milliseconds compared to cloud deployment. Therefore, the deployment of a Hyperledger-based solution could improve performance at different levels: the generation time of contextual access tokens and the transmission time of these tokens.

What can be added in this section is that the cost associated with each transaction with Ethereum is a last element that might make a Hyperledger-based implementation preferable to an Ethereum-based implementation. Indeed, with Ethereum, “gas” is used to measure the amount of work required to complete tasks such as transactions verification. Any operation related to identity verification and security of the system could involve a transaction fee of several cents (conversion between the amount of Ethereum gas and USD) [47]. On the contrary, with Hyperledger, a private blockchain, the cost of a transaction is zero.

### 5.3. Security Analysis

Implementing as-a-service context-aware authorization management in the Edge IoT networks may face several security threats. In this section, we analyze the security of our proposal by checking some properties.

#### 5.3.1. Contextual Access Tokens Security

The proposed contextual access tokens are secured to resist several attacks, including eavesdropping, modification (forging the content of a valid token or changing the content of a token during transfer), token replay, and privileges escalation. Indeed, tokens are encrypted and signed by the blockchain after generation, then transmitted to the client over a TLS secured channel. Encryption ensures the confidentiality of the token during transfer. Signing the token allows the client to authenticate the origin of the token, i.e., to ensure that the issuer of the token is the authorization blockchain. It also enables the client to ensure that the token has not been altered during the transfer. The token signature also allows the resource server to ensure that the decentralized authorization server issued the token. Thus, these mechanisms allow mitigating eavesdropping, token modification, and identity theft attacks during the transfer of the token from the blockchain to the client and from the client to the resource server.

Contextual access tokens are also protected against replay and use in invalid contexts. The context identity of a contextual access token enables the context-aware security service to validate the context. The token’s proof of possession (PoP) allows the context-aware security service to verify the token’s use by a legitimate client. When an already used token is presented to the context-aware security service, it invalidates the token. If the token’s context does not match the user’s context, the authorization request will be rejected. Besides, the proposed system mitigates privileges escalation attacks. In this sense, the tokens are generated and signed by the blockchain. It is almost impossible for a malicious user to modify a previously generated token without altering the authorization token signatures and escalate their privileges.

However, our system is vulnerable to a typical real-life attack on blockchain: private key theft [48]. When an attacker can access a user’s private keys, he can take ownership of user application authorizations or discard the user by deriving a new public key. That is why we considered the user’s identity protection in this paper threat model. User identity protection has not been considered in this work.

#### 5.3.2. Authorization Server Security

The authorization server is a critical element of the ACE-OAuth standard framework. It performs all the authorization management operations such as access and refresh tokens delivering and revocation. As mentioned in the introduction, the authorization server can constitute a single point of failure. Moreover, an attacker can compromise the server and deliver valid access tokens. Therefore, the risks associated with central authorization management are numerous.

In our proposition, decentralization improves authorization server robustness. Indeed, generating non-authorized access tokens will only be possible if 51% of the network’s miners are compromised [49]. For Bitcoin, the most widely used crypto-currency, the number of miners is currently equal to one million. Compromising more than 51% of one million machines seems very complex. Therefore, the decentralized architecture offers a higher level of robustness. We can also add that the level of availability allowed by this decentralized architecture is also higher. Indeed, even if an adversary takes control of a node and makes it unavailable, it will still be possible for clients to communicate with another node to access the authorization service. Therefore, our proposition is more secure and robust, failure tolerant, and brings more confidence in authorization management in the IoT.

#### 5.3.3. User and Clients Privacy

The proposed system protects the user’s privacy. First, as mentioned in Section 4, it is based on the ACE-OAuth framework. Then, our system allows the user to authorize applications (other users) without revealing his identity and credentials. In addition, the use of blockchain technology enables our solution to leverage one of the intrinsic advantages of the blockchain: the pseudonymization of the users’ identity. Indeed, for each user of the blockchain, a unique pseudonym is randomly generated. This user will then use this pseudonym to communicate with the blockchain. Therefore, this is the first step to strengthen users’ privacy: the user uses identifiers that cannot be directly linked to him. Besides, many works like [50,51], were interested in developing solutions that guarantee user intractability (unlinkability). In other words, the proposed solutions ensure that two transactions (requests) issued by the same user cannot be linked together through different mechanisms such as hiding the sender identity or using multiple addresses for each sender. Therefore, the combination of these two ideas (pseudonymization, untraceability) will guarantee a high level of privacy protection for the users of our service.

## 6. Conclusions

In this paper, we introduced a blockchain-enabled decentralized context-aware authorization management architecture “as a service” in IoT. This architecture solves the trust problem of the authorization server of the ACE framework by introducing the blockchain. This extension enables decentralized context-aware authorization management in the IoT. The “as a service” design allows its integration into any context-aware security service for the IoT. It also allows its implementation in new network architectures such as the edge IoT networks with very low latency, and supports the user’s mobility. With contextual access tokens, resource servers will only grant access to a resource if the user’s context allows it. The evaluation results proved the proposed architecture feasibility and efficiency compared to the classical ACE framework. The results also proved that the authorization server decentralization is acceptable and has a low impact on the overall latency of the authorization flow.

Nevertheless, this work presents limitations. First, our proposed system is limited by one of blockchain’s significant drawbacks—the private key protection problem. On the other hand, the resource server was tested on a single type of device. It would be interesting to evaluate performance on other IoT devices, for example, on Arduino MKR Wifi 1010 or ESP8266 Node MCU, and see how they can resist to the attacks (token tampering, side channel attacks, token forgering). Then, the experiments were done locally. Testing the orchestration and dynamic placement of the context-aware security service and blockchain nodes in a real-world Edge IoT infrastructure would confirm the results.

As for future work, we plan to implement our solution with an improved and dynamic zero-knowledge authentication protocol designed for IoT. We are also working on a use case and its deployment (orchestration, dynamic placement) in an edge infrastructure of a 5G network to evaluate its performances.

## Figures and Tables

**Table 1:** Comparison of the authorization management systems studied.

Work	Sec. T.	C. A. T.	D. T. M.	De. AM.	AS. I.	E. C.	Evaluated
[15]	No	Yes	No	No	No	No	No
[6]	Yes	No	No	No	No	No	Yes
[16]	Yes	No	No	No	No	No	Yes
[1]	No	No	No	No	No	Yes	No
[17]	Yes	No	yes	Yes	No	No	Yes
[19]	No	No	No	Yes	No	No	Yes
[21]	No	No	No	Yes	No	No	Yes
[20]	No	No	No	Yes	No	No	Yes
[23]	No	Yes	No	Yes	No	No	No
[22]	No	Yes	No	Yes	No	No	Yes
[8]	Yes	Yes	No	No	No	No	Yes
Our	Yes	Yes	Yes	Yes	Yes	Yes	Yes

Abbreviations: Sec. T., secure token; C.A.T., contextual access token; D.T.M., dynamic token management; De. AM., decentralized authorization management; AS. I., as a service integration; E.C., edge computing.

**Table 2:** User smart contract functions.

Functions	Can Be Called by
Generate contextual access token	Context-aware security service
Authorize a client	Context-aware security service
Request a contextual access token	Client
Upgrade a contextual access token	Client
Check token context	Context-aware security service
Revoke a contextual access token	Context-aware security service
Revoke a client	Context-aware security service

## Data Availability

The data that support the findings of this study are available from the corresponding author upon reasonable request.
